# Case Report of Malignant Hyperthermia in the Emergency Department

**DOI:** 10.5811/cpcem.1402

**Published:** 2023-05-26

**Authors:** Mitchell McMurray, Austin Sowers, Raymond Orthober, Martin Huecker

**Affiliations:** University of Louisville School of Medicine, Department of Emergency Medicine, Louisville, Kentucky

**Keywords:** case report, malignant hyperthermia

## Abstract

**Introduction:**

Malignant hyperthermia (MH) is a rare but deadly condition that may be encountered in the emergency department (ED). This report highlights a case of a patient who initially presented for acute agitation with hypertension and tachycardia and provides explanation for how to manage MH.

**Case Report:**

A 44-year-old male presented to the ED with altered mental status, eventually requiring intubation with etomidate and succinylcholine. Despite being afebrile initially, the patient developed a rectal temperature of 105.3° Fahrenheit (F) with significantly elevated arterial carbon dioxide levels after intubation. The treating team initiated cooling measures and dantrolene, leading to a positive outcome.

**Conclusion:**

Clinicians should strive for expeditious recognition of MH and treatment with an updated institutional protocol.

## INTRODUCTION

Malignant hyperthermia (MH) describes a condition of a hypermetabolic response to anesthetic gases (eg, halothane and sevoflurane) or the depolarizing muscle relaxant succinylcholine.^1^ Although rare clinically (estimated incidence of 1 in 10,000 to 1 in 250,000 anesthesia procedures), the genetic abnormality may occur as frequently as one in 3,000 individuals.^1^ The earliest signs are tachycardia, rise in end-tidal carbon dioxide (EtCO_2_), and muscle rigidity with hyperthermia usually occurring later.^1^ Left untreated, MH leads to cellular hypoxia (metabolic acidosis), rhabdomyolysis (hyperkalemia), myoglobinuria (with acute renal failure), cardiac arrythmias, and usually death.^1^

Patients susceptible to MH have a defective calcium channel (the ryanodine receptor) in the sarcoplasmic reticulum membrane.^2^ The mutation in this receptor (traced to the ryanodine receptor 1 gene) results in uncontrolled intracellular calcium release with exposure to triggering agents.^2^ Rapid adenosine triphosphate depletion occurs, and the muscle membrane degrades.^2^ Inheritance is an autosomal dominant pattern and genetic testing using muscle biopsy confirms the diagnosis.^2^ Of note, MH is significantly more common in pediatric patients (about 1 in 100,000 adults and 1 in 30,000 in children).^2^ As a result, succinylcholine is sparingly used for rapid sequence intubation (RSI) in pediatrics.^2^

Most people remain unaware of their susceptibility to MH until the disease occurs. Although rarely used, both the caffeine-halothane contracture test (CHCT) and in vitro contracture test (IVCT) can reliably diagnose MH.^3^,^4^ Both measure the contracture response of freshly biopsied muscle to different levels of caffeine and halothane.^4^ Improved recognition and testing capabilities have decreased mortality from 70% 30 years ago to less than 5% today.^1^,^5^,^6^

## CASE REPORT

A 44-year-old male with a past medical history of depression and chronic back pain presented to the emergency department (ED) with altered mental status. According to his wife, he had been in normal health early that morning. Later in the morning he began to have an abnormal gait “like Frankenstein,” but retained normal speech and mentation. His wife returned home to find the patient with his extremities convulsing, diaphoretic, and eyes open but unresponsive. The patient had reportedly consumed alcohol the evening before but did not use recreational drugs. Emergency medical services administered naloxone 2 milligrams (mg) intravenous (IV), lorazepam 2 mg IV, and one liter of normal saline IV without improvement in mental status. The ground crew called a flight ambulance who transported the patient to our Level I trauma center.

Upon presentation to the ED, the patient had tachycardia in the 130s but was normotensive, afebrile, and had normal oxygenation saturation on room air. On physical exam, he was extremely diaphoretic and tremulous, but he moved all extremities spontaneously. His eyes (pupils 5 millimeters [mm] and reactive) opened spontaneously, he withdrew from painful stimuli, and he made incomprehensible sounds (Glasgow Coma Score 10). The patient had clear breath sounds bilaterally, no abdominal distention or tenderness, and no obvious signs of trauma. The initial chest radiograph (CXR) showed no acute findings; point-of-care lab testing was significant only for mild lactic acidosis, 1.8 millimoles per liter (mmol/L) (reference range: 0.5–2.2 mmol/L). The treatment team ordered two 2-mg doses of midazolam in an attempt to control agitation, but the patient remained combative. Given the patient’s declining mental status and need for emergent clinical workup, the treatment team made the decision to perform RSI.

Intubation was performed successfully using etomidate and succinylcholine followed by propofol infusion for sedation. After CXR confirmed endotracheal tube placement, the patient was transported for computed tomography (CT). Non-contrast head CT showed no acute findings and initial laboratory work was significant for acute kidney injury with creatinine of 2.31 mg per deciliter (mg/dL) (reference range: 0.7–1.3 mg/dL) and a leukocytosis of 27,400 thousand per microliter (uL) (reference range: 4,000–11,000 thousand per uL) with 84% neutrophils (55–70%). Urine and serum toxicology studies, troponin level, and coronavirus disease 2019 polymerase chain reaction (PCR) were all negative.

The team provided further fluid resuscitation, empiric antibiotic coverage for meningitis, and prepared for a lumbar puncture. Prior to the procedure, the patient developed rigid muscles diffusely and felt warm to the touch. Rectal temperature was 105.3° F, a significant increase from his triage temperature of 98.9° F approximately 90 minutes prior. His arterial carbon dioxide (pCO2) also increased to 65.3 mm of mercury (Hg) from 46.6 mm Hg (reference range: 35–45 mm Hg) immediately post-intubation despite no changes in ventilator settings. With concern for MH, the team began active external cooling measures, initiated our MH protocol, and consulted the anesthesia service for dantrolene administration. Fifty minutes after dantrolene administration, the patient’s rectal temperature decreased to 101.3° F. The patient’s rigidity resolved and pCO2 decreased to 31.2 mm Hg two hours after dantrolene. The patient was admitted to the intensive care unit (ICU) under the anesthesia service.

*CPC-EM Capsule*

What do we already know about this clinical entity?

*Malignant hyperthermia (MH) is a rare, life-threatening condition that occurs during anesthesia procedures involving succinylcholine or anesthetic gases*.

What makes this presentation of disease reportable?

*We diagnosed and treated a case of MH in the ED, where this condition is not typically described*.

What is the major learning point?

*Emergency departments should have an institutional protocol in place and access to dantrolene for management of MH*.

How might this improve emergency medicine practice?

*Succinylcholine is a common paralytic used in the ED. We hope this case encourages clinicians to be prepared to recognize and treat MH*.

The patient received dantrolene for 24 hours while inpatient. Neurology service consultation led to an electroencephalogram, which showed no seizure activity. The ICU team added acyclovir to cover for possible herpes simplex virus (HSV) infection and performed a lumbar puncture. Cerebrospinal fluid analysis was consistent with viral meningitis. HSV-1 and -2 PCR testing were negative, and the team discontinued antimicrobials on hospital day three. After full diagnostic workup, the final diagnosis made by the ICU team for his original presentation of altered mental status was determined to be viral meningitis. The patient passed spontaneous breathing trials, began following commands, and was subsequently extubated. He was discharged on hospital day four in good condition with planning for a CHCT through the University of Minnesota. The patient was lost to follow up; so, it is unclear whether he received confirmatory testing.

## DISCUSSION

Malignant hyperthermia is a well described and rare condition that emergency physicians (EP) may encounter, although exact incidence of ED presentations is not described in the literature. While EPs will likely not directly use anesthetic gases, MH has a well-known association with succinylcholine, which is commonly used by EPs for RSI.^7^ A database analysis and systematic review of over six million perioperative cases determined that use of succinylcholine without volatile anesthetics triggered 24 MH cases.^7^ Another study examined the risk of MH based on whether or not a patient had received succinylcholine.^8^ They determined that for all cases the relative risk was 19.6 for those with compared to without succinylcholine.^8^ It is clear that any clinician using anesthetic gases or depolarizing paralytic agents should understand the presentation and management of MH. Although there is no known screening test for MH, it would be prudent for EPs to gather information on family history of MH or prior anesthesia complications before intubation, if possible, given the high mortality.^9^ If a patient with a known personal or family history of MH is being treated in an ED, clinicians and staff should be notified immediately so that use of succinylcholine is avoided.

Our patient initially presented for acute agitation with hypertension and tachycardia, a common presentation in the ED setting. This presentation yielded a broad differential diagnosis list: substance-induced (i.e., methamphetamine, cocaine, phencyclidine), serotonin syndrome, neuroleptic malignant syndrome; encephalitis; and malignant catatonia, among others. After RSI, the patient had rapid increases in temperature, pCO2, and muscle rigidity, leading to suspicion for MH.

Diagnosis of MH is made by a combination of clinical findings and laboratory testing.^4^ Signs and symptoms suggestive of MH are unexplained increased EtCO_2_ concentration, muscular rigidity, muscle breakdown, combined metabolic and respiratory acidosis, hyperthermia, cardiac arrhythmia, etc.^4^ An international panel of 11 experts on MH created a clinical grading scale to estimate the likelihood of an MH event.^4^ The score assigns point totals to different physiologic markers and also takes into account family history.^4^ A diagnosis of MH is likely when scores are greater than 20 and is almost certain if greater than 50.^4^ Our patient’s score exceeded 50 when using this scale.

The current gold standard for diagnosing MH is the CHCT or IVCT, which is mainly used in Europe.^4^ The IVCT has a sensitivity of 99.0% and a specificity of 93.6%, while CHCT demonstrates a sensitivity of 97% and a specificity of 78%.^4^ It is unclear in the literature how often suspected patients receive confirmatory testing, but it is estimated to be low given the rarity of MH and the limited diagnostic centers that perform the testing. There are currently only four available centers for CHCT testing in North America listed on the Malignant Hyperthermia Association of the United States (MHAUS) website.^10^ Overall treatment goals of MH include discontinuation of trigger agents, active cooling measures, hyperventilation, and administration of dantrolene.^1^ A postsynaptic muscle relaxant, dantrolene acts as an antagonist to muscle ryanodine receptors.^2^ When indicated, dantrolene is given as a bolus initial dose (2.5 mg per kilogram [kg]) followed by maintenance infusion (1 mg/kg) for 24–48 hours with careful observation for reappearance of symptoms.^2^ The figure demonstrates a possible algorithm for management of MH.

The MHAUS recommends initiating dantrolene infusion less than 10 minutes after diagnosis.^10^ Clinicians must act quickly to mobilize the product because most patients require multiple vials mixed with sterile water prior to administration.^10^ In our case, the patient received dantrolene in a timely fashion with significant improvement in symptoms and a positive outcome. Dantrolene availability varies due to cost, storage requirements, and difficulty in administration.^10^

The best way to be prepared for a case of MH in the ED is having a detailed protocol in place. Our EPs relied on the institutional protocol throughout the treatment course. At a minimum, the MHAUS recommends facilities that stock and administer any triggering medication to have dantrolene available in case MH occurs.^11^ Many facilities have an MH cart that includes dantrolene and interventions to treat the effects of the hypermetabolic condition such as sodium bicarbonate, insulin, calcium, amiodarone, etc.^10^ Lack of rapid identification and treatment of the disease process leads to patient death due to severe hyperthermia and acidosis.^2^ Patients will require admission to the ICU, as mortality is up to 5% even with proper treatment.^2^

Throughout management of the case, the treatment team used the MH hotline (1-800-644-9737). This resource affords clinicians with continuous access to experienced staff that gives medical oversight and could mean the difference between life and death for patients.

## CONCLUSION

Although more commonly described in the anesthesia literature, malignant hyperthermia can occur in the ED and must be diagnosed and managed without delay for favorable outcomes. Patients should be screened when feasible for prior anesthesia complications, and all patients should be closely monitored following succinylcholine administration. It is strongly recommended that EDs have an institutional protocol and clinicians have access to dantrolene for management of MH.

## Figures and Tables

**Figure:**
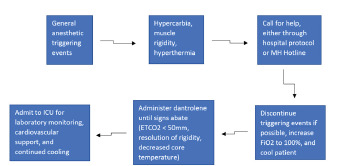
Diagnosing and treating suspected malignant hyperthermia in the emergency department *MH*, malignant hyperthermia; *ICU*, intensive care unit; *ETCO2*, end-tidal carbon dioxide; *Fi02*, fraction of inspired oxygen.
